# Endoplasmic reticulum resident oxidase ERO1-Lalpha promotes hepatocellular carcinoma metastasis and angiogenesis through the S1PR1/STAT3/VEGF-A pathway

**DOI:** 10.1038/s41419-018-1134-4

**Published:** 2018-10-30

**Authors:** Shikun Yang, Chao Yang, Fei Yu, Wenbing Ding, Yuanchang Hu, Feng Cheng, Feng Zhang, Bugao Guan, Xuehao Wang, Ling Lu, Jianhua Rao

**Affiliations:** 1 Hepatobiliary/Liver Transplantation Center, the First Affiliated Hospital of Nanjing Medical University; Key Laboratory on Living Donor Liver Transplantation of National Health and Family Planning Commission of China, Nanjing, 210029 China; 2 Department of General surgery, People’s Hospiltal of Jinhu, Jinhu, Huan’an China

## Abstract

Mounting evidence demonstrates that expression of ERO1α, an endoplasmic reticulum (ER)-resident oxidase, is a poor prognosis factor in a variety of human cancers. However, the clinical relevance of ERO1α and its molecular mechanisms underlying tumor progression have not been determined for hepatocellular carcinoma (HCC). ERO1α expression levels in HCC tissues and cells were detected by quantitative real-time PCR and western blotting. ERO1α shRNAs and overexpression vector were transfected into HCC cells to downregulate or upregulate ERO1α expression. In vitro and in vivo assays were performed to investigate the function of ERO1α in invasion, metastasis, and angiogenesis of HCC. We found high ERO1α expression in HCC tissues and cells that was significantly associated with metastasis and poor clinicopathologic features of vascular invasion, advanced Edmondson Grade, and TNM stage. Loss-of-function and gain-of-function studies showed that ERO1α prompted migration, invasion, epithelial–mesenchymal transition (EMT), and angiogenesis of HCC cells both in vitro and in vivo. Further studies verified a positive correlation between ERO1α and S1PR1, upregulated in metastatic HCC tissues compared with HCC tissues without metastasis. *S1PR1* knockdown markedly diminished the effects of ERO1α on HCC cell migration, invasion and vascular endothelial growth factor (VEGF) expression. Most importantly, ERO1α knockdown significantly repressed the death of HCC xenograft mouse models by reducing tumor distant metastasis, and host angiogenesis by suppressing the expression of S1PR1, p-STAT3, and VEGF-A in HCC cells. Our findings suggest that ERO1α is significantly correlated with reduced survival and poor prognosis, and promotes HCC metastasis and angiogenesis by triggering the S1PR1/STAT3/VEGF-A signaling pathway. ERO1α might be a novel candidate in HCC prognosis and therapy.

## Introduction

Hepatocellular carcinoma (HCC) is the fifth most prevalent malignancy and the second leading cause of cancer-associated deaths worldwide^1^, with incidence rates increasing rapidly^2^. Although hepatectomy or liver transplantation is the most effective treatment for long-term survival, the overall survival (OS) for patients with HCCs remains unsatisfactory due to relapse and metastasis after surgery^3^. In addition, some patients have early metastasis, which prevents hepatectomy or liver transplantation^4^. Thus, exploring the deeper mechanisms leading to HCC invasion and metastasis is urgent for finding new prognostic and therapeutic strategies.

ERO1α, a hypoxia-inducible endoplasmic reticulum (ER)-resident oxidase^5,6^, is activated following ER stress under abnormal conditions, including hypoxia, metabolic disorders, and oxidative stress. ERO1α is essential for the formation of disulfide bonds in protein synthesis^7^. A recent study indicated that ERO1α activation coupled with glutathione transport preserves ER redox poise^8^. Under abnormal conditions commonly seen in tumors, proteins are unfolded or misfolded in the ER lumen, provoking an evolutionarily conserved adaptive response called ER stress^9^. Sustained activation of the ER stress response endows malignant cells with greater tumorigenic, metastatic, and drug-resistant capacity and impedes development of protective anticancer immunity^10^. ER stress-related ERO1α contributes to cells coping with ER stress as a result of an adaptive homeostatic response^11^. ERO1α is overexpressed and is a poor prognosis factor in various kinds of cancers including breast, colon, and pancreatic cancer^12–14^. However, the clinical relevance of ERO1α and the molecular mechanisms underlying tumor progression have yet to be determined in HCC.

Sphingosine-1-phosphate (S1P), a multifunctional lipid mediator, regulates cell growth, survival, differentiation, lymphocyte trafficking, vascular maturation, permeability, and angiogenesis^15,16^. S1P receptor 1 (S1PR1) is one of five G protein-coupled receptors for S1P, and is crucial for the retention of lymphocytes in secondary lymphoid organs^16,17^. S1PR1 has key functions in tumor metastasis and angiogenesis^18,19^, and maintains persistent STAT3 activation by regulating both tumor cells and tumor-infiltrating myeloid cells^20^. Prior study found that the S1PR1-STAT3 signaling pathway is crucial for myeloid cell colonization at future metastatic sites^21^. Therefore, we were interested in detecting the expression of and determining the relationship between ERO1α and S1PR1 in HCC.

We found that ERO1α expression was upregulated in human HCC tissues compared with adjacent tissues. This expression was involved in reducing survival and poor prognosis in HCC. Mechanistically, we showed that ERO1α prompted angiogenesis, migration, and invasion of hepatoma cells via the S1PR1/STAT3/VEGF-A signaling pathway both in vitro and in vivo. These results highlighted the dual role for ERO1α in promoting tumor metastasis.

## Results

### ERO1α expression is significantly upregulated in HCC tissues and cell lines

**Fig. 1:**
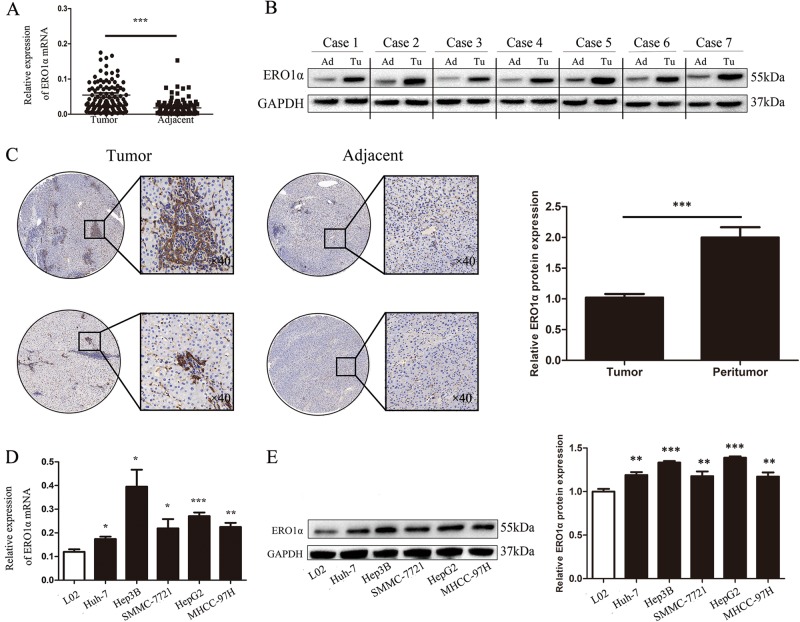
ERO1α expression is significantly upregulated in HCC tissues samples and cell lines. **a** ERO1α mRNA and **b** protein in HCC tumor tissues compared with adjacent nontumor tissues. **c** Representative IHC staining of ERO1α in HCC and adjacent tissues. **d** ERO1α mRNA and **e** protein in HCC cell lines and normal L02 cells by RT-qPCR and western blotting. *P < 0.05, **P < 0.01, ***P < 0.001

To explore the function of ERO1α in HCC development, we investigated levels of ERO1α mRNA and protein in tumor tissues and matched adjacent nontumor tissues from 114 patients with HCC. We observed higher ERO1α mRNA and protein levels in tumor tissues compared with adjacent nontumor tissues (Fig. 1a, b). Typically, ERO1α-positive staining was observed in HCC tumor tissues with ERO1α-negative or weak staining in adjacent nontumor tissues from patients with HCC (Fig. 1c). Similar results were shown in The Cancer Genome Atlas (TCGA) database, and we found that ERO1α expression was significantly higher in high-grade HCC compared to low-grade HCC or normal tissues (Fig. S1A,B). In addition, we checked ERO1α expression in L02 normal liver cell line and five human HCC cell lines including HepG2, Hep3B, SMMC-7721, MHCC-97H, and Huh-7, and found significantly increased ERO1α mRNA levels in HCC cell lines (Fig. 1d). Consistent with this result, we further found that ERO1α protein expression was upregulated in HCC cells (Fig. 1e). These data indicated that expression of ERO1α is significantly upregulated in HCC tissues and HCC cell lines.

### **Upregulated ERO1α expression predicts poor prognosis and correlates with HCC metastasis**

**Fig. 2:**
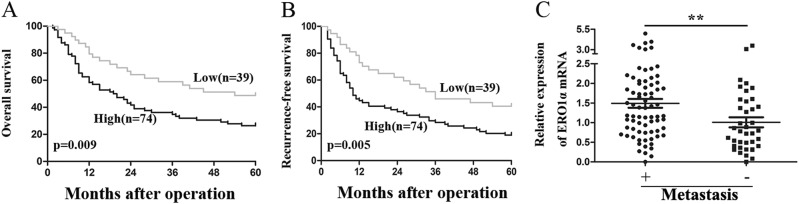
Upregulated ERO1α predicts poor prognosis and correlates with metastasis for HCC patients. **a** Kaplan–Meier association of higher ERO1α levels with shorter OS and (**b**) RFS. **c** ERO1α in HCC with and without metastasis by RT-qPCR. Absence (−) (*n* = 40) or presence (+) (*n* = 71) of venous invasion (tumor thrombus in veins of adjacent nontumor tissues or portal vein), lymph node metastasis (*n* = 2), or both (*n* = 1). Central horizontal line, mean; error bars, SD. **P < 0.01

To investigate the clinical significance of ERO1α expression in HCC, patients were divided into high and low expression groups by ERO1α staining intensity. We analyzed the relationship between clinicopathologic features and ERO1α expression in 114 pairs of HCC tissues. High levels of ERO1α were significantly associated with tumor vascular invasion (*P* = 0.003), tumor pathologic stage (*P* = 0.009), and tumor TNM stage (*P* = 0.034) (Table 1). No significant association was found between ERO1α expression and age, gender, tumor size, AFP: alpha fetoprotein; HBV: hepatitis B virus infection, or liver cirrhosis. We further evaluated the prognostic value of ERO1α for patients with HCC using Kaplan–Meier survival analysis. HCC patients with tumors with high ERO1α expression had decreased 5-year OS and shorter recurrence-free survival (RFS) than patients with tumors with low ERO1α expression (*P* = 0.009, Fig. 2a; *P* = 0.005, Fig. 2b), consistent with that in TCGA database (Fig.S1C). Furthermore, higher ERO1α levels were significantly associated with a higher presence of metastasis (Fig. 2c), suggesting that ERO1α upregulation may have contributed to HCC progression by promoting tumor metastasis. Thus, our data suggest that ERO1α might represent a novel indicator of poor HCC prognosis and be an HCC progression marker.

**Table 1:** Clinicopathological correlation of ERO1α expression in HCC

Variables	ERO1α expression		*χ* ^2^	*P* value
	High	Low		
All cases	65	39		
Age (years)			0.354	0.552
≤50	33	21		
>50	22	18		
Gender			0.178	0.673
Male	47	26		
Female	28	13		
HBs antigen			2.080	0.149
Absent	12	12		
Present	53	27		
Liver cirrhosis			1.303	0.254
With	45	31		
Without	20	8		
AFP (ng/ml)			1.410	0.235
≤20	24	10		
>20	41	29		
Tumor size			1.667	0.197
≤3 cm	29	20		
>3 cm	46	19		
Vascular invasion			8.816	**0.003**
Absent	21	22		
Present	54	17		
TNM stage			4.486	**0.034**
I–II	25	21		
III–IV	50	18		
Edmondson grade			6.769	**0.009**
I–II	27	24		
III–IV	48	15		

The bold number means statistically significant.

### ERO1α promoted cell migration and invasion of HCC cells in vitro

**Fig. 3:**
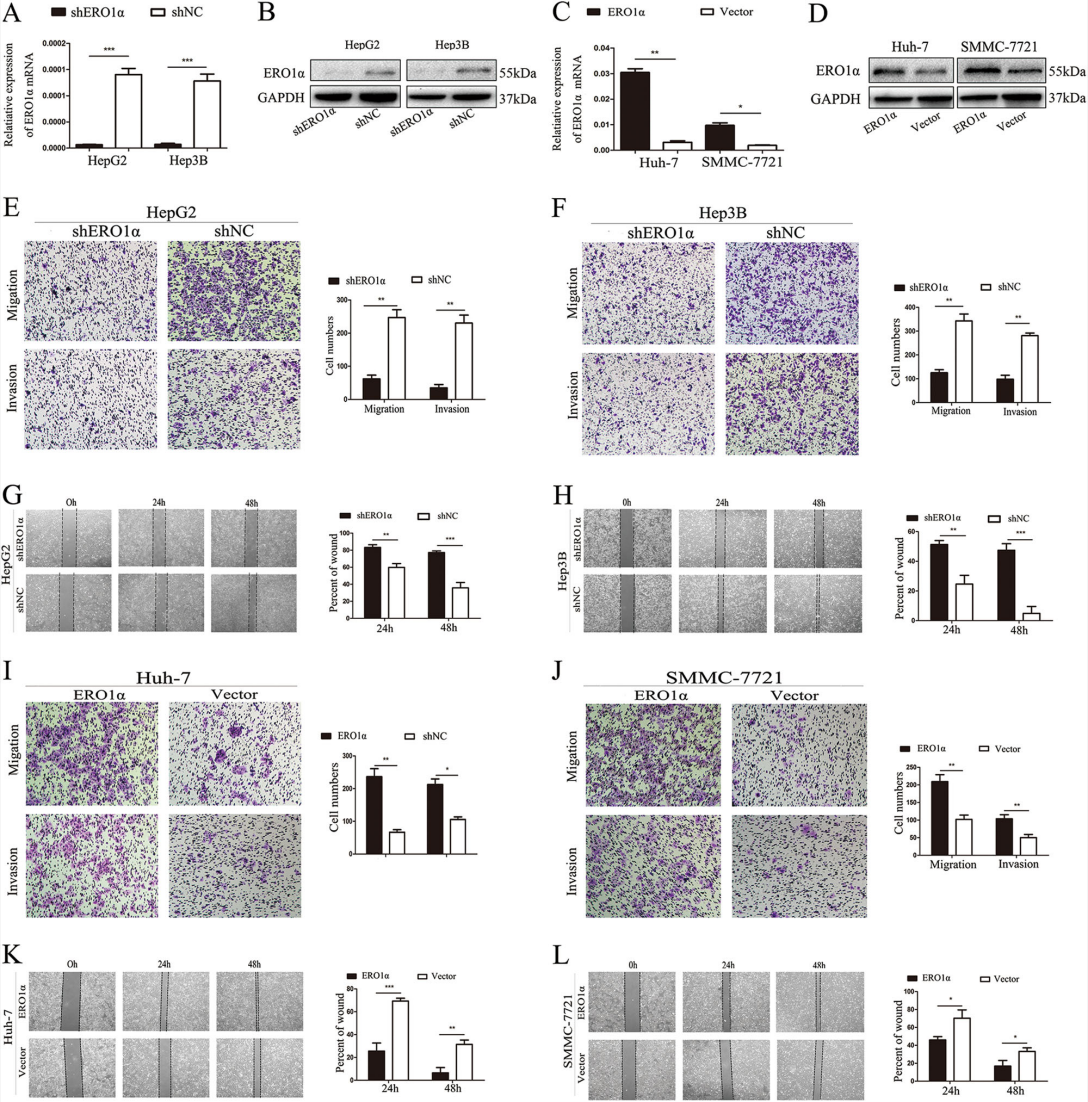
ERO1α promoted cell migration and invasion of HCC cells in vitro. **a** HepG2 and Hep3B cells transfected with shRNA targeting ERO1α were used as shERO1α. Cells transfected with empty lentiviral vectors were negative controls (shNC). ERO1α was detected with RT-qPCR and **b** western blotting. **c** Huh-7 and SMMC-7721 cells were transfected for ERO1α overexpression. Cells transfected with empty lentiviral vectors were used as controls. ERO1α was analyzed by RT-qPCR and **d** western blotting after transfection. **e**, **f**, **i**, **j** Transwell assays for cell migration and invasion and **g**, **h**, **k**, **l** wound-healing assays. Cell migration and invasion were quantified as cell numbers. All experiments were performed three times. Data are mean ± SD. *P < 0.05, **P < 0.01, ***P < 0.001

To assess the biological function of ERO1α in HCC, we investigated the effect of ERO1α knockdown on migration and invasion of HCC cells. We knocked down ERO1α expression in HepG2 and Hep3B cells, which have a relatively high level of ERO1α expression, using specific short hairpin RNAs (shRNAs) targeting ERO1α. As confirmed using quantitative real-time reverse transcription polymerase chain reaction (RT-qPCR) and western blotting, ERO1α expression was significantly downregulated by shRNA in HepG2 and Hep3B cells compared with control cells (Fig. 3a, b). Similarly, ERO1α overexpression by lentivirus transduction was confirmed in Huh-7 and SMMC-7721 cells (Fig. 3c, d). Transwell assays showed that inhibition of ERO1α significantly reduced cell migration and invasion of HepG2 and Hep3B cells (Fig. 3e, f), whereas ERO1α overexpression increased cell migration and invasion in Huh-7 and SMMC-7721 cells (Fig. 3i, j). A similar result was observed in cell wound-healing assays (Fig. 3g, h, k, l). These results indicate that ERO1α promoted cell migration and invasion of HCC cells in vitro.

### ERO1α promoted HCC cell epithelial–mesenchymal transition

**Fig. 4:**
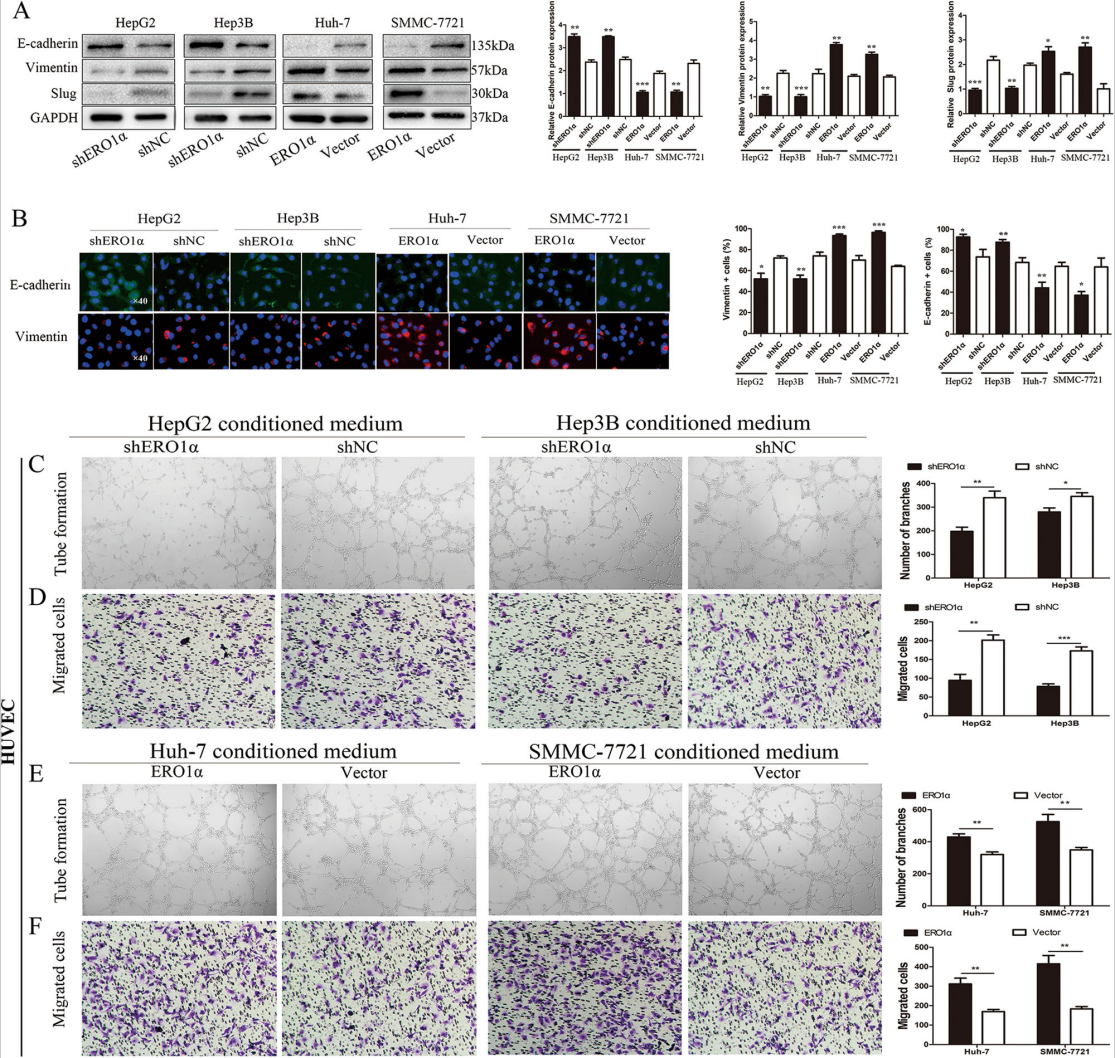
ERO1α promoted HCC cell epithelial–mesenchymal transition (EMT) and its ability to promote HUVEC tube formation. **a** Western blotting and **b** immunofluorescent staining for levels of EMT-related proteins with 4′,6-diamidino-2-phenylindole (blue) to identify nuclei. Scale bars = 50 μm. **c**, **e** Tube-formation assays with HUVECs in indicated conditioned media. Numbers of branches were calculated using Image Pro Plus 6. **d**, **f** Endothelial recruitment assays with HUVECs performed for each group. Cell migration was quantified as cell numbers. All experiments were performed three times. Data are mean ± SD. *P < 0.05, **P < 0.01, ***P < 0.001

Given that cancer cells of epithelial origin could metastasize by transforming into cells with a mesenchymal phenotype (epithelial–mesenchymal transition; EMT), during EMT, epithelial cells gradually losing their connection to the basement membrane, degrading the extracellular matrix, can increase invasive potential. Importantly, EMT is characterized by decreased expression of cell adhesion molecules such as E-cadherin and increased transformation of cytokeratin into vimentin. Thus, EMT is important in cancer cell invasion and metastasis, we therefore tested to determine whether ERO1α was involved in regulation of the EMT process of cancer cells. We examined typical EMT proteins. Western blotting indicated that after ERO1α downregulation in HepG2 and Hep3B cells, E-cadherin levels increased while vimentin and Slug levels were reduced. In contrast, overexpression of ERO1α showed the opposite results in SMMC-7721 and Huh-7 cells (Fig. 4a). In addition, EMT immunofluorescence revealed a similar phenomenon (Fig. 4b). These data suggest that ERO1α promotes cell migration, invasion, and EMT in HCC cells.

### **ERO1α enhanced the capability of tumor cell to promote** human umbilical vein endothelial cell **tube formation**

Angiogenesis is indispensable for cancer cell growth and metastasis. To elucidate the effect of ERO1α on HCC angiogenesis, in vitro tube formation and endothelial recruitment assays were performed. Tube-formation assays with human umbilical vein endothelial cells (HUVECs) used different tumor conditioned medium (TCM). Compared with control cells, the shERO1α group decreased HUVEC tube formation. The group overexpressing ERO1α overexpression enhanced the capability of tumor cells to prompt tube formation by HUVECs (Fig. 4c, e).

We used endothelial recruitment assays to investigate the effects of ERO1α on HUVEC migration. HUVEC migration was increased with TCM derived from Huh-7 and SSMC-7721 cells transfected to overexpress ERO1α compared with TCM from control cells. In contrast, HUVEC migration was suppressed with TCM derived from HepG2 and Hep3B-shERO1α cells (Fig. 4d, f). Taken together, these results suggest that ERO1α enhanced the ability of tumor cells to promote HUVEC tube formation.

### ERO1α augmented expression of vascular endothelial growth factor-A

**Fig. 5:**
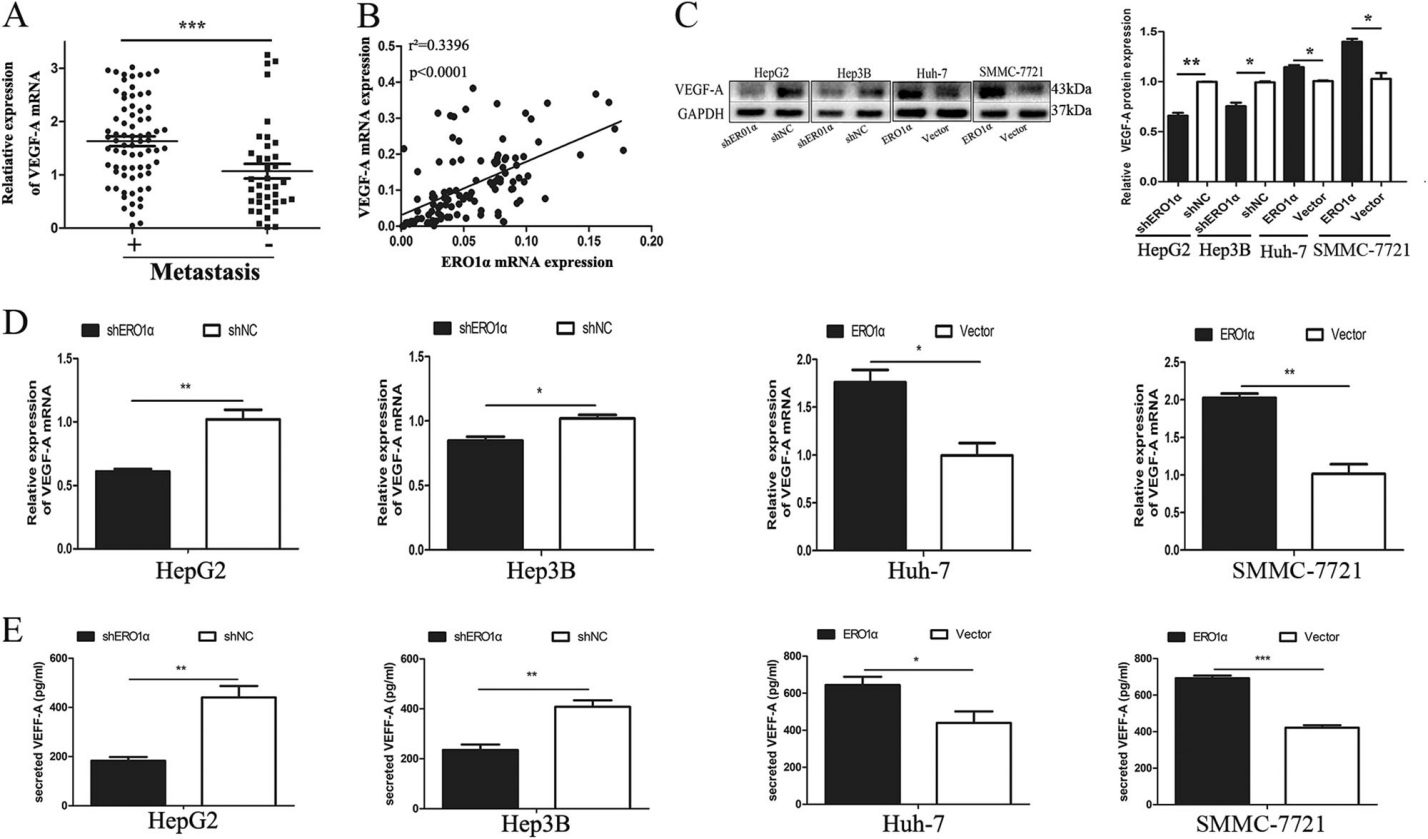
ERO1α augmented expression of VEGF-A. **a** VEGF-A expression analyzed for 114 pairs of HCC tissues from the HCC cohort described in Fig. 2. Central horizontal line, mean; error bars, SD. **b** Positive correlation between ERO1α and VEGF-A levels in HCC specimens. **c** VEGF-A protein by western blots. **d** RT-qPCR for levels of VEGF-A mRNA in tumor cells. **e** ELISA for VEGF-A concentration in TCM. Data are mean ± SD. *P < 0.05, **P < 0.01, ***P < 0.001

Vascular endothelial growth factor-A (VEGF-A) is the most important angiogenic factor influencing vasculature and angiogenesis^22^. We detected VEGF-A expression in HCC tissues and found that metastatic HCC samples had higher levels of VEGF-A (Fig. 5a) and its upregulation positively correlated with ERO1α overexpression (Fig. 5b). We used HepG2-shERO1α, HepG2-shNC, Hep3B-shERO1α, Hep3B-shNC, SMMC-7721-ERO1α, SMMC-7721-vector, Huh-7-ERO1α, and Huh-7-vector cells to test VEGF-A levels. Compared with HepG2-shNC and Hep3B-shNC cells, VEGF-A mRNA decreased by approximately 40% in HepG2-shERO1α and 20% in Hep3B-shERO1α cells (Fig. 5d). VEGF-A mRNA levels in SMMC-7721-ERO1α and Huh-7-ERO1α cells were approximately double the level in homologous scramble cells. Similarly, VEGF-A protein levels were decreased in HepG2-shNC cells and Hep3B-shERO1α cells, and increased in SMMC-7721-ERO1α cells and Huh-7-ERO1α cells, compared with corresponding negative control cells (Fig. 5c). We used enzyme-linked immunosorbent assays (ELISAs) to detect secreted VEGF-A protein in the TCM of the cell lines. ERO1α knockdown decreased levels of secreted VEGF-A levels, while ERO1α overexpression augmented secreted VEGF-A levels (Fig. 5e). These results suggest that ERO1α augmented VEGF-A expression in HCC.

### **S1PR1/STAT3/VEGF-A signaling is critical for ERO1α-mediated promotion of migration, invasion, and angiogenesis**

**Fig. 6:**
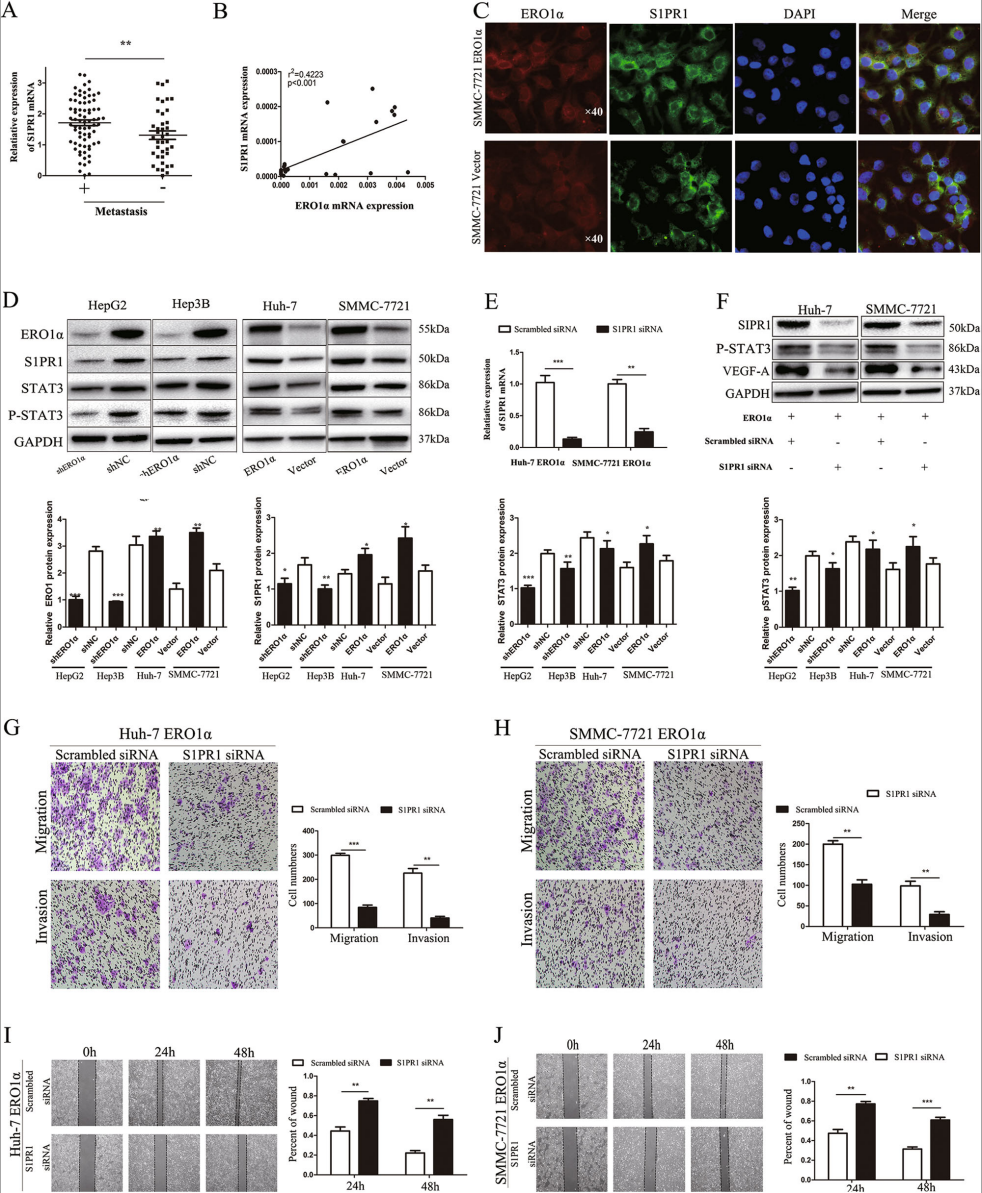
S1PR1/STAT3/VEGF-A signaling is critical for ERO1α-mediated promotion of migration, invasion, and angiogenesis. **a** S1PR1 expression was analyzed in HCC tissues from the HCC cohort with or without metastasis described in Fig. 2. **b** Positive correlation between ERO1α and S1PR1 levels in HCC specimens. **c** Immunofluorescent staining images for ERO1α and S1PR1 in SMMC-7721-ERO1α and SMMC-7721-Vector cells. 4′,6-Diamidino-2-phenylindole (blue) was used to identify nuclei. Scale bars = 50 μm. **d** Indicated molecules were evaluated by western blots with four HCC cell lines, using GAPDH as the loading control. **e** RT-qPCR and **f** western blots for S1PR1 and VEGF-A in indicated cells. **h**–**j** Rescue experiments for ERO1α-overexpressing cells with S1PR1 silencing. Downregulated S1PR1 counteracted Huh-7 and SMMC-7721 cell migration and invasion that was enhanced by ERO1α overexpression. Cell migration and invasion were quantified as cell numbers. All experiments were performed three in triplicate. Data are mean ± SD. *P < 0.05, **P < 0.01, ***P < 0.001

Previous studies reported that S1PR1, one of five G protein-coupled receptors for S1P, could maintain persistent STAT3 activation in tumorigenesis and is essential for tumor metastasis and angiogenesis^16–19^. To better understand the mechanisms by which ERO1α functioned in metastasis and angiogenesis, we examined S1PR1 expression in HCC and adjacent normal tissues. Metastatic HCC samples displayed higher levels of S1PR1 (Fig. 6a), in agreement with the positive correlation between ERO1α expression and HCC metastasis. Meanwhile, linear correlation analysis verified the positive correlation between ERO1α and S1PR1 (Fig. 6b). Furthermore, immunofluorescence showed that ERO1α and S1PR1 mainly colocalized in the cytoplasm of SMMC-7721 cells, and ERO1α overexpression can obviously enhance S1PR1 expression levels (Fig. 6c). Therefore, we hypothesized that ERO1α upregulation might contribute to S1PR1 upregulation in HCC. To test this hypothesis, we examined S1PR1 expression and analyzed the correlation between ERO1α and S1PR1 levels in ERO1α knockdown or ERO1α-overexpressing cells and control cells. S1PR1 expression was downregulated in ERO1α knockdown cells, while upregulated S1PR1 was detected in ERO1α-overexpressing cells (Fig. 6d).

Given that S1PR1-STAT3 signaling is crucial for tumor metastasis^20,21^ and our results showed that VEGF-A, downstream of the STAT3 pathway, was correlated positively with ERO1α expression, we futher determined expression of STAT3 and phospho-STAT3 (p-STAT3) in ERO1α-knockdown or ERO1α-overexpressing cells using western blots (Fig. 6d). STAT3 and p-STAT3 expression decreased in ERO1α-knockdown cells. In ERO1α-overexpressing cells, STAT3 and p-STAT3 expression increased. To confirm that S1PR1 was essential for ERO1α-induced metastasis, we silenced S1PR1 in ERO1α-overexpressing cells using specific small interfering RNA (siRNA). S1PR1 expression was detected using RT-qPCR and western blotting (Fig. 6e, f). Transwell (Fig. 6g, h) and wound-healing assays (Fig. 6i, j) showed that silencing S1PR1 significantly inhibited migration and invasion in ERO1α-overexpressing cells. Tube-formation assays and endothelial recruitment assays with HUVECs demonstrated that silencing S1PR1 significantly inhibited tube formation and migration of HUVECs (Fig. S1D). Cell migration was quantified as cell numbers. Silencing S1PR1 significantly reversed the expression of p-STAT3 and VEGF-A in ERO1α-overexpressing cells (Fig. 6f). Collectively, these results showed that ERO1α promoted migration, invasion, and angiogenesis of HCC through S1PR1/STAT3/VEGF-A signaling and S1PR1 was essential for ERO1α-induced metastasis in HCC.

### S1PR1/STAT3/VEGF-A contributes to ERO1α-mediated distant metastasis and angiogenesis in a HCC xenograft model

**Fig. 7:**
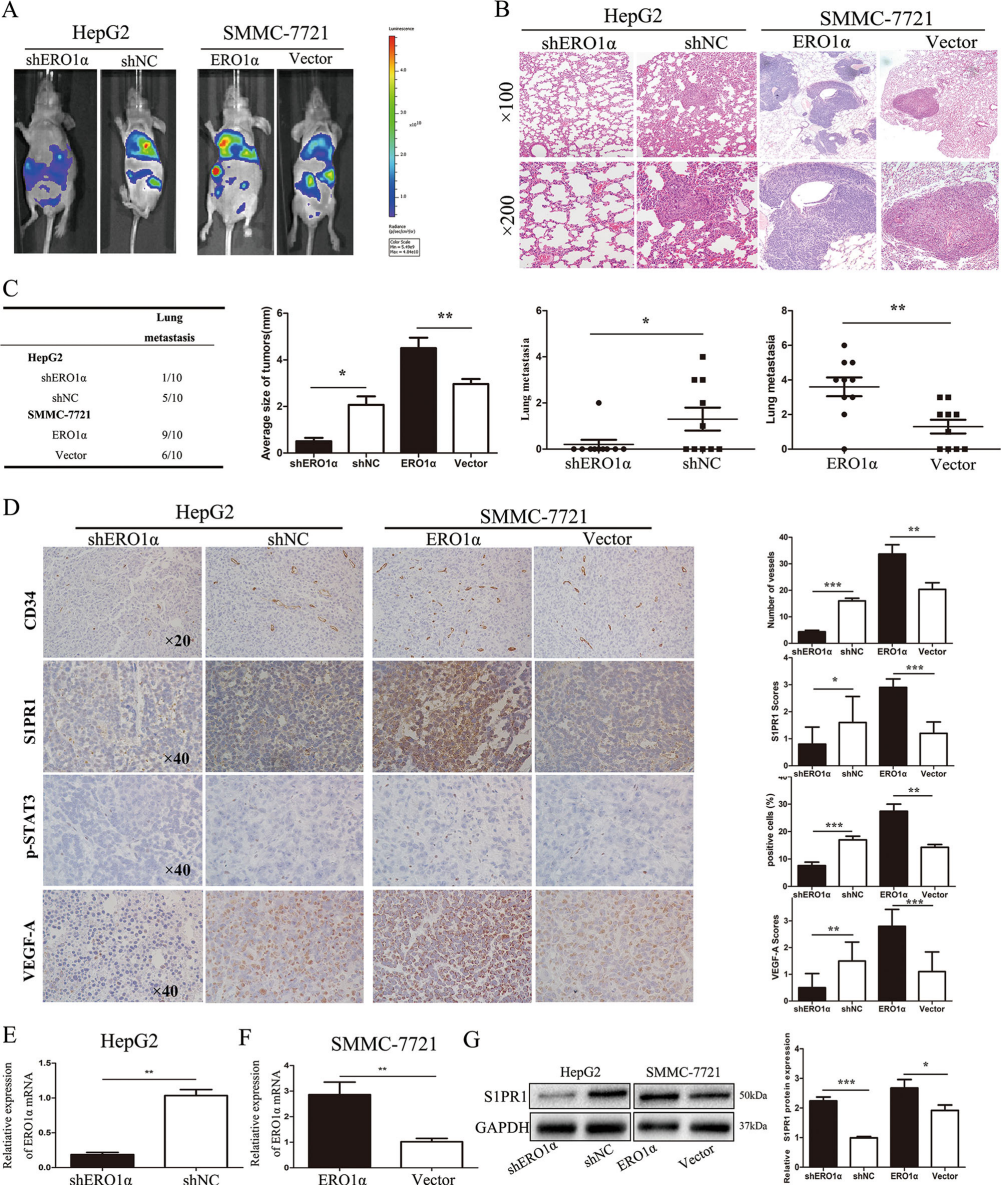
S1PR1/STAT3/VEGF-A pathway contributes to ERO1α-mediated distant metastasis and angiogenesis in an HCC xenograft model. **a** Photographs of tumors by an IVIS Imaging System. Representative luciferase signals were captured for each group 7 weeks after injecting indicated cells. **b** HE-stained lung sections. **c**, **d** ERO1α expression in metastatic tumors derived from nude mice measured by RT-qPCR. **e** S1PR1 protein in metastatic tumors derived from nude mice measured by western blots. **f** CD34, S1PR1, p-STAT3, and VEGF-A analyzed in tumor tissues by immunohistochemistry. Blood vessels were stained using anti-CD34, and positively stained vessels were counted in five areas per slide to determine maximum number of microvessels; 10 slides per experiment. Results are mean ± SD. *P < 0.05, **P < 0.01, ***P < 0.001

To investigate the effect of ERO1α on HCC migration and invasion in vivo, SMMC-7721-ERO1α, SMMC-7721-vector, HepG2-shERO1α, and HepG2-shNC cells were inoculated into nude mice through tail veins. Lung metastasis nodules were examined 35 days after injection. In vivo fluorescence imaging revealed that cells overexpressing ERO1α exhibited more distant metastases than the negative control group (Fig. 7a). Lung metastasis nodules from mice injected with cells were confirmed by hematoxylin and eosin (HE) staining. The number of nodules in lungs from mice injected with ERO1α-overexpressing cells was significantly increased compared with empty vector-transfected groups. ERO1α knockdown significantly reduced distant metastasis (Fig. 7b, c). These results suggest that ERO1α overexpression promoted HCC cells migration and invasion in vivo, consistent with our results in vitro.

We isolated several lungs that have higher HCC metastatic tropism and detected S1PR1, p-STAT3, VEGF-A, and CD34 expression using immunohistochemistry. The number of CD34-positive microvessels increased with SMMC-7721-ERO1α, whereas HepG2-shERO1α decreased the number of CD34-positive microvessels compared with shNC (Fig. 7d). Similar results were observed for S1PR1 and VEGF-A expression in tumors (Fig. 7d). We also validated ERO1α expression in metastatic tumors derived from nude mice with RT-qPCR. ERO1α levels increased in the SMMC-7721-ERO1α-treated group while the decreased level was shown in the HepG2-shERO1α-treated group (Fig. 7e, f). Additionally, S1PR1 expression was determined with western blotting. The level of S1PR1 protein was decreased in the HepG2-shERO1α-treated group, whereas increased in the SMMC-7721-ERO1α-treated group (Fig. 7g).

## Discussion

HCC is a highly lethal malignancy due to active angiogenesis and frequent metastasis. Tumor metastasis involves molecular-level changes that disrupt and modify a series of tumor cell–cell and cell–extracellular matrix interactions^23,24^. Tumor hypoxia, a characteristic of the tumor microenvironment, especially in solid tumors such as HCC^25^, exerts pronounced effects on malignant progression and metastatic spread of cancers^26^. Tumor cells take advantage of factors induced by hypoxic circumstances to thrive. As the expression of ERO1α is induced under hypoxic conditions^6^, we hypothesized that ERO1α had a vital function in the progression process of HCC. Previous studies showed that ERO1α is overexpressed in multiple kinds of cancers, and is involved in tumor biological procession and immune escape^13,14,27^, however, its impact on HCC remains unknown.

In this study, we found that ERO1α was observably upregulated in HCC tissues compared with adjacent nontumor tissues. Analysis of clinical data revealed that increased levels of ERO1α correlated with poor prognostic features such as tumor vascular invasion, advanced tumor pathologic stage, and tumor TNM stage. Compared with low ERO1α expression, HCC patients with high ERO1α expression had markedly decreased 5-year OS and shorter RFS. These results suggest that ERO1α is an independent predictor of prognosis in HCC, and ERO1α may contribute to HCC metastasis. Furthermore, our results reveal that ERO1α prompted the migration, invasion, angiogenesis, EMT, and lung metastasis of hepatoma in vitro and in vivo, suggesting that ERO1α upregulation was involved in HCC progression. In line with our findings, the prognostic value of ERO1α elucidated in this study was concordant with the main findings for other cancers^13,14,28^. For instance, Kutomi et al. also found that ERO1α is highly expressed in breast cancer cell lines and clinical specimens and promotes cell migration and angiogenesis^13^. Another report showed that ERO1α promotes cancer progression through modulation of integrin-β1 modification in colorectal cancer^14^. To our knowledge, this is the first report that ERO1α acts as a promoter in HCC, establishing a novel connection between ER stress-signaling pathways and cancer progression and suggesting that ERO1α may be a prognostic marker for HCC.

HCC is a highly vascularized tumor and angiogenesis is vital for tumorigenesis and progression. The migration and tube formation of HUVECs are important processes mirrored in tumor angiogenesis^29^. ERO1α is reported to be pivotal for VEGF production via hypoxia-inducible factor 1^30^. Other studies indicated that ERO1α facilitate angiogenesis by augmentation of VEGF production in breast cancer^28^. Our findings revealed that ERO1α overexpression in HCC cells enhanced the capability of tumor cells to promote HUVEC migration and tube formation in vitro. Conversely, ERO1α depletion decreased microvessel density in vivo. Further experiments indicated that ERO1α promoted tumor angiogenesis and positively correlated with VEGF-A expression via the S1PR1/STAT3/VEGF-A pathway, suggesting that ERO1α may be a novel therapeutic target for inhibiting tumor angiogenesis. We also found that ERO1α significantly decreased E-cadherin expression, while increasing the levels of vimentin and Slug proteins, indicating that EMT was enhanced. Takei et al. revealed that ERO1α knockout diminished EMT via integrin activation in colorectal carcinoma. However, the specific mechanism between ERO1α and EMT in HCC remains to be further defined.

S1PR1, one of the five G protein-coupled receptors for S1P, is crucial for the retention of lymphocytes of secondary lymphoid organs^16,17^ and tumor angiogenesis and metastasis^18,19^. A study identified S1PR1 as a critical target for reducing acquired and environment-mediated drug resistance in neuroblastoma^31^. S1P is a potent bioactive sphingolipid metabolite that regulates cell growth, survival, differentiation, lymphocyte trafficking, vascular integrity, and cytokine and chemokine production that are important for inflammation and immune responses^15,16^. S1P is regarded as a crucial moderator in cancer^32–34^. Studies report that S1PR1 induces persistent activation of STAT3, and STAT3, a transcription factor for S1PR1, induces S1PR1 expression in a positive feedback loop for maintaining persistent STAT3 activation in tumor cells and the tumor microenvironment for malignant progression^20,21^. Our results showed that ERO1α and S1PR1 had a positive correlation relationship, and expression of STAT3, p-STAT3, and VEGF-A was consistent with ERO1α levels. Furthermore, silencing S1PR1 reversed the migration and invasion abilities of ERO1α-overexpressing HCC cells. Hence, we concluded a novel molecular mechanism in which ERO1α promoted the metastasis and angiogenesis of HCC through S1PR1/STAT3/VEGF-A signaling, suggesting that reagents targeting the pathway would be beneficial for inhibiting tumor metastasis and angiogenesis. However, the specific mechanisms between ERO1α and S1PR1-STAT3 signaling has not yet been fully understood. Whether as reported by Tanaka et al. in VEGF, ERO1α play a role in HCC via oxidative protein folding of S1PR1 remains further study.

In conclusion, our study revealed that ERO1α is associated with poor prognosis and promotes the migration, invasion, EMT, and angiogenesis of HCC both in vitro and in vivo via the S1PR1/STAT3/VEGF-A signaling pathway. Thus, strategies designed to treat ERO1α as a novel prognostic indicator and potential therapeutic target for HCC may be promising.

## Materials and methods

### Human tissue specimens

HCC tissues and corresponding adjacent nontumor tissues were obtained from 114 patients who underwent surgical resection of primary HCC between 2010 and 2012 in the First Affiliated Hospital of Nanjing Medical University (Nanjing, China) with informed consent. Patients were selected using the following criteria: (a) confirmed pathologic diagnosis; (b) no distant metastases; (c) curative liver resection; (d) no preoperative radiotherapy or chemotherapy; and (e) availability of detailed follow-up and clinicopathologic data. This project and the study protocol were approved by the Ethics Committee of Nanjing Medical University (Nanjing, China).

### Cell lines and cultures

The human HCC cell lines MHCC-97H, SMMC-7721, Huh-7, HepG2, and Hep3B, L02 immortalized liver cell line, and HUVECs were obtained from the Cell Bank of Chinese Academy of Science (Shanghai, China). All cells were cultured in Dulbecco’s modified Eagle’s medium (DMEM, Life Technologies, Carlsbad, CA, USA) supplemented with 10% fetal bovine serum (FBS; Life Technologies) and 1% penicillin/streptomycin in a 37 °C incubator with 5% CO2. For cell harvesting and passaging, we used 0.25% trypsin with 0.01% EDTA and phosphate-buffered saline (PBS).

### RNA extraction and RT-qPCR

Total RNA was extracted from surgically resected HCC or adjacent nontumor tissues and HCC cell lines using TRIzol (Invitrogen, Carlsbad, CA, USA) and reverse transcribed to cDNA with an RT kit (Vazyme, Nanjing, China) according to the manufacturer’s protocol. For RT-qPCR, we used a ChamQ Universal SYBR qPCR Master Mix (Vazyme) according to the product manual on an ABI 7900 Fast Real-Time PCR system (Applied Biosystems, Foster City, CA, USA). β-actin was used as the endogenous control. Each experiment was performed in triplicate. Primers were:

ERO1α forward: 5′-GGCTGGGGATTCTTGTTTGG-3′ and reverse: 5′-AGTAACCACTAACCTGGCAGA-3′;

S1PR1 forward: 5′-TTCCACCGACCCATGTACTAT-3′ and reverse: 5′-GCGAGGAGACTGAACACGG-3′;

VEGF-A forward: 5′-AGGGCAGAATCATCACGAAGT-3′ and reverse: 5′-AGGGTCTCGATTGGATGGCA-3’;

β-actin forward: 5′-TGACGTGGACATCCGCAAAG-3′ and reverse: 5′-CTGGAAGGTGGACAGCGAGG-3’.

### **Western blots**

Concentrations of proteins extracted from cell lines or tumor tissues were determined by bicinchoninic acid assay kits (Beyotime, Shanghai, China) following the manufacturer’s instructions. Proteins were resolved by 10% sodium dodecyl sulfate-polyacrylamide gel electrophoresis and transferred to a polyvinylidene fluoride membranes (Bio-Rad, Hercules, CA, USA). After blocking with 5% skim milk powder at room temperature for 2 h, membranes were incubated with primary antibody (1:1000) overnight at 4 °C. Appropriate secondary antibody (1:2000) was added before incubation for 2 h at 37 °C. Protein band intensities were measured using Image Lab software. GAPDH was used as the internal control. Western blots were quantified using Image Pro Plus version 6. Primary antibodies were from rabbits against S1PR1, VEGF-A (Abcam, Cambridge, UK), STAT3, p-STAT3, E-cadherin, vimentin, Slug, and GAPDH (Cell Signaling Technology, Danvers, MA, USA); and from mice against ERO1α (Santa Cruz Biotechnology, Santa Cruz, CA, USA).

### ERO1α **knockdown and overexpression**

Commercially available lentiviral-mediated ERO1α knockdown vector or the negative control (shERO1α/shNC) and lentiviral-mediated overexpressing ERO1α vector or scrambled lentiviral construct (ERO1α/Vector) were designed and produced (GenePharma Co. Ltd Shanghai, China). All vectors were verified by sequencing. ERO1α shRNA and the ERO1α-overexpressing lentivirus were transfected into cells according to the manufacturer’s instructions. After incubating in medium containing lentiviral particles for 24 h, transfection medium was replaced with normal medium. Target cells were treated with puromycin (Sigma-Aldrich, St. Louis, MO, USA) (3 μg/mL) for 14 days to select cells with stably integrated lentiviral vectors. ERO1α expression was detected using RT-qPCR and western blotting.

### S1PR1 knockdown

A specific siRNA for S1PR1 and a nonspecific duplex oligonucleotide as a negative control were synthesized by GenePharma Co. Ltd. Using Lipofectamine 2000, siRNAs were transfected into cells following the manufacture’s protocol. S1PR1 expression was determined with RT-qPCR and western blotting.

### Migration and invasion assays

Using a 6.5-mm chamber with 8 μm pores (Corning, Corning, NY, USA), migration and invasion assays were performed with HCC cells. For migration assays, cells were added into upper chambers with noncoated membranes. For invasion assays, cells were placed on top chamber inserts precoated with 100 μL 2% Matrigel (BD Biosciences, Franklin Lakes, NJ, USA). A total of 5 × 10^4^ cells suspended in 200 μL serum-free DMEM were added into upper chambers for both assays, with 500 μL DMEM with 10% FBS added to the lower chamber. After 24 h at 37 °C, cells that migrated into or invaded the underside of membranes were fixed with 4% paraformaldehyde, stained with 0.5% crystal violet for 30 min at 37 °C, washed with PBS, and counted. At least six random fields of a phase-contrast microscope (Olympus, Tokyo, Japan) were observed at ×100 magnification, and counted for each chamber. Experiments were performed three times in triplicate.

### Wound-healing assays

Cells (5 × 10^5^) were added to six-well plates and allowed to grow until 90% confluent. Using a plastic pipette tip, a vertical wound was made in the monolayer, and detached cells were removed. The distance between the wound sides was photographed and measured after 0, 24, and 48 h at 37 °C. For each experiment, at least three scratched fields were recorded.

### Preparation of TCM

Tumor cells transfected with shERO1α or shNC and ERO1α or vector were cultured as above. When cells reached 80% confluency, they were cultured in media free of serum for an additional 24 h. Supernatant was collected after centrifugation at 2000 × *g* for 20 min at 4 °C and TCM was stored at −80 °C.

### Enzyme-linked immunosorbent assay

The concentration of VEGF-A in TCM was detected with ELISA kits (Dakewei, Shenzhen, China) according to the manufacturer’s instructions. The concentration of VEGF-A in 100 μL TCM was determined. Standard curves were created using serial dilutions of recombinant human VEGF-A included in each assay.

### HUVEC tube formation assays

For tube-formation assays, HUVECs were suspended at 2 × 10^4^ cells/mL in TCM 50 μL cell suspensions added to wells of µ-Slide angiogenesis plate (ibidi, Martinsried, Germany) precoated with 10 μL Matrigel (BD Biosciences), DMEM was the negative control. After 6 h at 37 °C, tube formation was observed and photographed using an inverted microscope (Olympus), The number of tube branches in each well was counted and calculated using Image Pro Plus.

### HUVEC recruitment assays

For recruitment assays, HUVECs were suspended at 2.5 × 10^4^ cells/mL in DMEM and 400 μL cell suspension added into upper chambers (Corning) as aforementioned. Lower chambers received 600 μL TCM. After 36 h at 37 °C, cells that migrated to the underside of membranes were fixed with 4% paraformaldehyde, stained with 0.5% crystal violet for 30 min at 37 °C, washed with PBS, and counted. At least six random fields of a phase-contrast microscope (Olympus) were observed at ×100 magnification and counted for each chamber.

### Immunohistochemistry

Tissues were fixed in 4% paraformaldehyde and embedded in paraffin. In citrate buffer (pH 6.0), slides were heated in an autoclave for 3 min for antigen retrieval. Slides were incubated with appropriate primary antibody (ERO1α from Santa Cruz; or S1PR1 from Abcam; or p-STAT3, VEGF-A, or CD34 from CST) overnight at 4 °C, followed by counterstaining with hematoxylin. The ERO1α expression status was graded by two independent observers as described by immunohistochemistry score: scoring was conducted based on the percentage of positive-staining cells: 0–5% scored 0, 6–35% scored 1, 36–70% scored 2, and more than 70% scored 3; staining intensity: no staining scored 0, weakly staining scored 1, moderately staining scored 2, and strongly staining scored 3. The final score was calculated using the percentage score × staining intensity score as follows: “0” for a score of 0–1, “1” for a score of 2–3, “2” for a score of 4–6, and “3” for a score of >6. In subsequent analyses, scores “0” and “1” were defined as the low group and scores “2” and “3” were defined as the high group scores.

### Immunofluorescence assays

Tumor cells were permeabilized with 0.1% Triton X-100 for 15 min and washed with PBS. Cells were blocked with PBS containing 5% bovine serum albumin for 1 h at room temperature. After treating with primary antibodies overnight at 4 °C, cells were rinsed with PBS and incubated with secondary antibody for 1 h at room temperature. Cells were counterstained with diamidino phenylindole and examined using a fluorescence microscopy (Leica Microsystems Imaging Solutions, Cambridge, UK). Antibodies were as aforementioned.

### Tumor xenograft models in nude mice

Female nude mice (BALB/C-nu/nu, 4–6 weeks old) were from the Laboratory Animal Resources Center of Nanjing Medical University (Nanjing, China). A total of 40 nude mice were randomly divided into four groups and HepG2-shERO1α/shNC, SMMC-7721-ERO1α/vector cells (2 × 10^6^ cells in 100 μL PBS) were injected into tail veins. After 5 weeks, mice from each group were humanely killed after anesthesia to obtain lung tissue for metastatic nodule evaluation and histopathological studies. Distant metastases were investigated using an in vivo fluorescence imaging system (Caliper life Sciences, Hopkinton, MA, USA) and HE staining. All experiments were approved by the Institutional Animal Care and Use Committee of Nanjing Medical University.

### Statistical analysis

GraphPad Prism 5 and SPSS v.22.0 software (SPSS Inc., Chicago, IL, USA) were used for statistical analyses. Data from at least three separate experiments were presented as mean ± SD. Student’s *t*-tests were used to evaluate differences between two groups. Qualitative data were evaluated with chi-square tests. OS and RFS rates after operation were calculated by the Kaplan–Meier method, and differences in survival curves were assessed by log-rank tests. Correlations were analyzed by Pearson correlation analysis. *P* < 0.05 was considered statistically significant.

## Electronic supplementary material

supplementary Fig. 1

supplementary Fig1 legend
