# Supplementary figures and images for: Endoplasmic reticulum resident oxidase ERO1-Lalpha promotes hepatocellular carcinoma metastasis and angiogenesis through the S1PR1/STAT3/VEGF-A pathway
Source: Cell Death Dis. 2018 Oct 30;9(11):1105. doi: 10.1038/s41419-018-1134-4 (PMC6207574; doi:10.1038/s41419-018-1134-4)

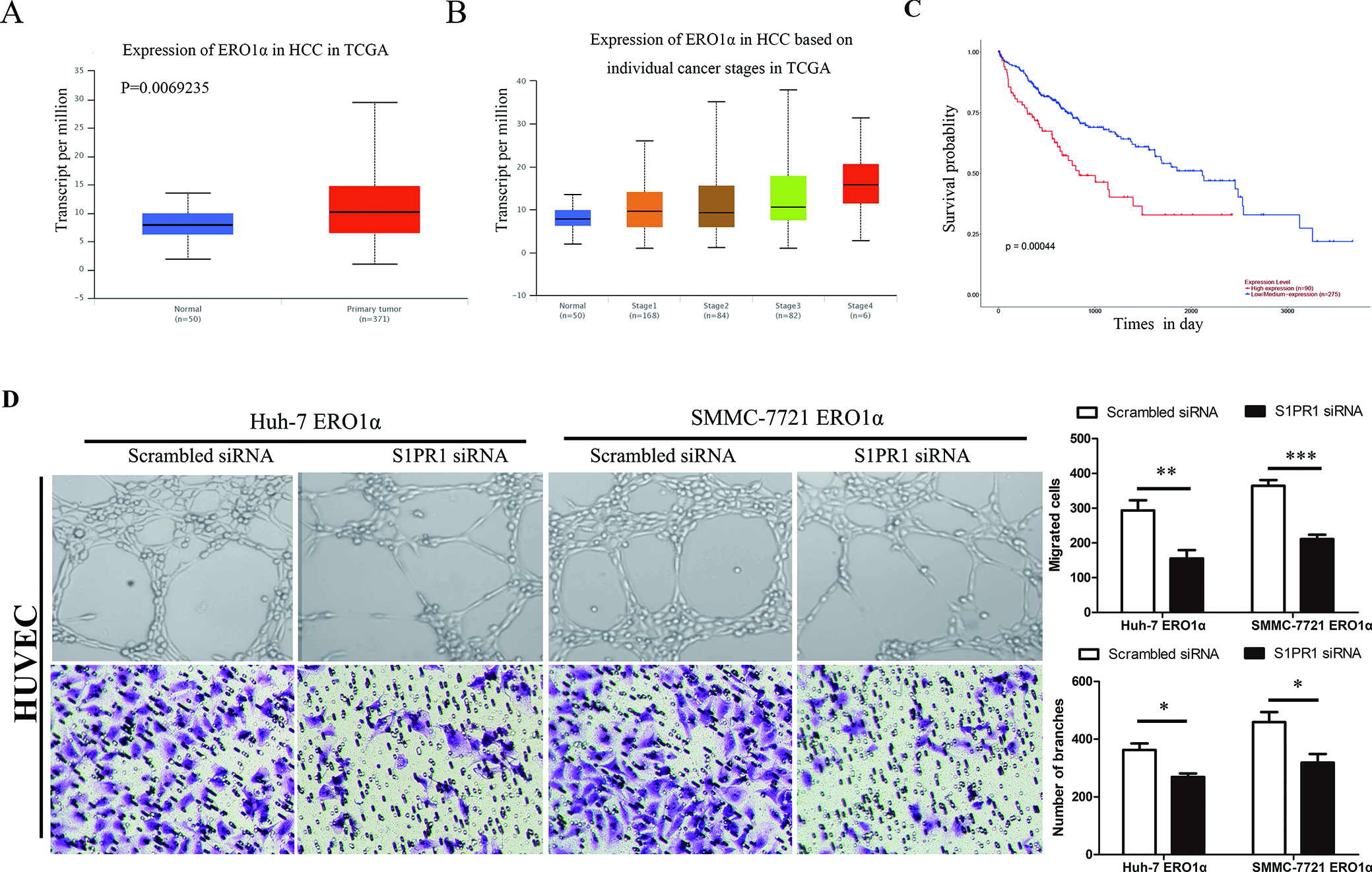

Supplement: Supplementary file 1 — supplementary Fig. 1 [file 41419_2018_1134_MOESM1_ESM.tif]
