# Supplementary material for: Endoplasmic reticulum resident oxidase ERO1-Lalpha promotes hepatocellular carcinoma metastasis and angiogenesis through the S1PR1/STAT3/VEGF-A pathway
Source: Cell Death Dis. 2018 Oct 30;9(11):1105. doi: 10.1038/s41419-018-1134-4 (PMC6207574; doi:10.1038/s41419-018-1134-4)
Supplement: Supplementary file 2 — supplementary Fig1 legend [file 41419_2018_1134_MOESM2_ESM.docx]

**Fig. S1**

Related information of ERO1α in TCGA database and rescue experiments (A)Comparison of ERO1α expression in HCC tissues and normal liver tissues in the TCGA database. (B)TCGA database showing increased ERO1α expression in high-grade HCC tissues compared with that in low-grade tissues and normal tissues. (C) Overall survival analysis of patients with high or low ERO1α expression in the TCGA database(D)Rescue experiments for ERO1α-overexpressing cells with S1PR1 silencing. Downregulated S1PR1 counteracted Huh-7 and SMMC-7721 cell ability to recruit HUVECs and promote HUVECs migration that was enhanced by ERO1α overexpression. Numbers of branches were calculated using Image Pro Plus 6. Cell migration was quantified as cell numbers. All experiments were performed three times. Data are mean ± SD. *P < 0.05, **P < 0.01, ***P < 0.001.
